# Beryllium^10^: a free and simple tool for creating and managing group safety data sheets

**DOI:** 10.1186/1758-2946-6-6

**Published:** 2014-03-20

**Authors:** Sven Kochmann

**Affiliations:** 1 Institute of Analytical Chemistry, Chemo- and Biosensors; University of Regensburg, Universitätsstr. 31, 93053 Regensburg, Germany

**Keywords:** Safety data sheet, Toxicological data, Globally Harmonised System (GHS), Laboratory hazards and risks, Laboratory safety

## Abstract

**Background:**

Countless chemicals and mixtures are used in laboratories today, which all possess their own properties and dangers. Therefore, it is important to brief oneself about possible risks and hazards before doing any experiments. However, this task is laborious and time consuming.

**Summary:**

Beryllium^10^ is a program, which supports users by carrying out a large part of the work such as collecting/importing data sets from different providers and compiling most of the information into a single group safety data sheet, which is suitable for having all necessary information at hand while an experiment is in progress. We present here the features of Beryllium^10^, their implementation, and their design and development criteria and ideas.

**Conclusion:**

A program for creating and managing of group safety data sheets was developed and released as open source under GPL. The program provides a fast and clear user-interface, and well-conceived design for collecting and managing safety data. It is available for download from the web page http://beryllium.keksecks.de.

## Background

Nowadays, many types of chemicals and mixtures are used in laboratories. Some of them are harmless and do not cause any problem. Most of them are dangerous if not handled with great care, though. Since each chemical possesses its very own properties and dangers, a vendor has to provide material safety data sheets (MSDSs) to inform users and purchasers about these properties and dangers. In Europe MSDSs are part of the regulations (EC) No 1907/2006 (REACH) and (EC) No 453/2010 [1,2], and there are similar regulations in other countries.

A MSDS contains multiple pages with no less than sixteen sections of data about one chemical [2]. The amount and presentation of data sometimes makes a MSDS very confusing. This is amplified by the fact that in contrast to the content the layout of a MSDS is not clearly specified. So, every vendor uses his own layout since there is no standard template. For common tasks such as syntheses more than one chemical usually is required and these chemicals frequently are purchased from different sources. Due to this it is laborious and time consuming to find and compile safety information such as disposal or first-aid. Additionally, particular dangers such as teratogenicity may be overlooked. Based on our own experience these are the reasons why the task of briefing oneself about dangers is neglected, not only by freshmen but by experienced researchers.

**Figure 1:**
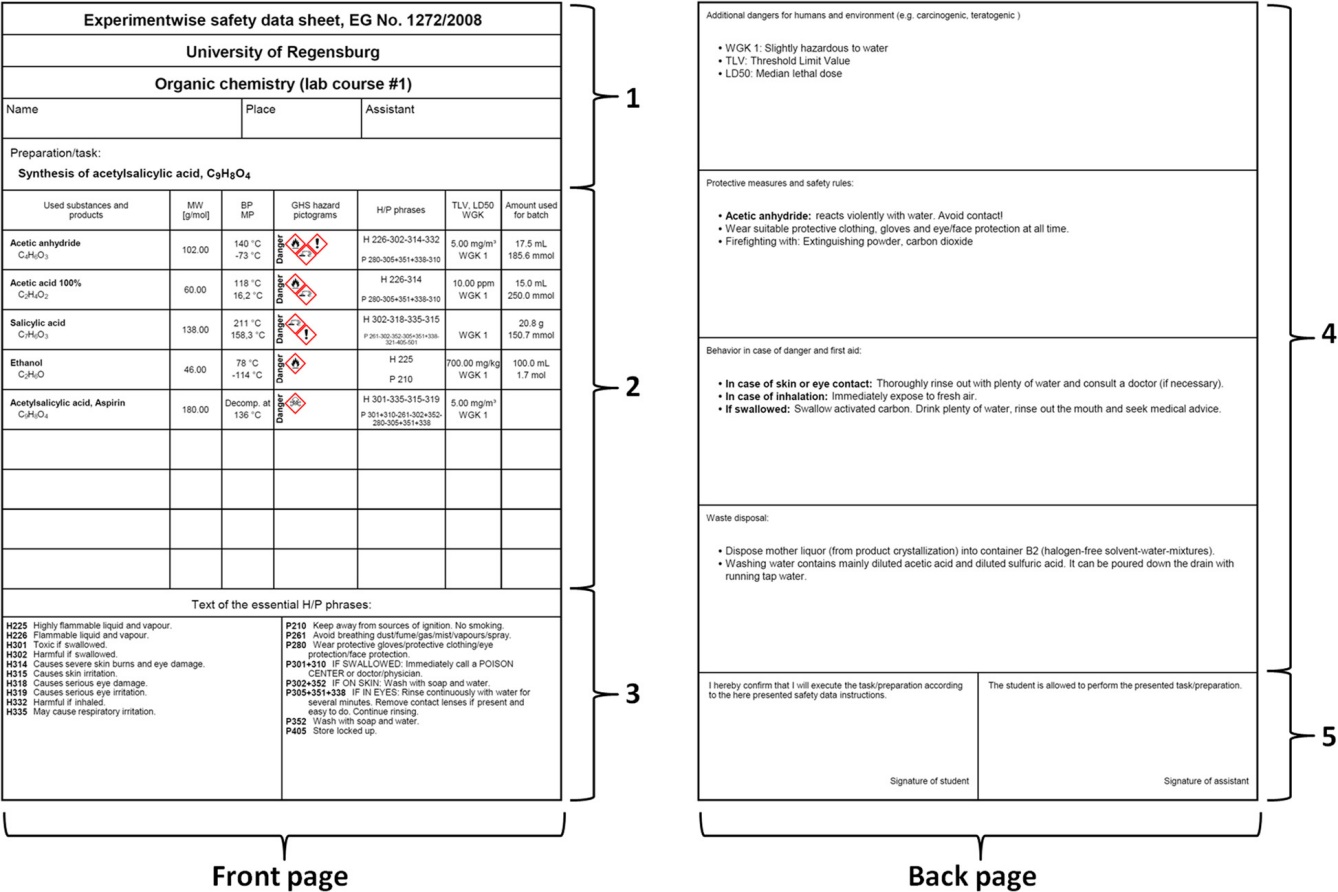
**Overview of an examplary group safety data sheet for the synthesis of Aspirin.1)** Head section with general information about the institute, user, and the task to be performed. **2)** List of substances needed for this task with compiled safety data. **3)** Text of essential and important H/P phrases. **4)** Summary information about dangers, safety rules, behavior in case of danger, and waste disposal for the whole task. **5)** Signatures of the user and the supervisor.

German students have to fill out so-called ‘group safety data sheets’ (GSDSs) for experiments (syntheses) they do in laboratory courses. A GSDS contains only the most important safety information for all chemicals required for one experiment on just two pages. It has to be signed by the student and the assistant/supervisor to confirm that the student is fully aware of the dangers before starting the experiment itself. An examplary GSDS with its different sections is displayed in Figure 1. There are two main benefits of this system: First, students are forced to deal with the dangers of chemicals. Secondly, their effort results in a clear overview of these dangers, which are always at hand during the experiment since it contains only two pages (equal one sheet of paper). This makes GSDSs not only very useful for students attending laboratory courses but also for day-to-day tasks, routines, and experiments in research laboratories.

When looking at the exemplary GSDS in Figure 1, one will notice the limited space of two pages. Limited space is a feature and the basic concept of a GSDS. It forces the author to focus on only the most important information available.

A GSDS is divided into different sections. The head section (1) provides five lines with general information about the institute/university, the course/department, the user, and the task or experiment, which the user wants to perform. Required substances and mixtures for this task are listed in the next block (2). The last block (3) on the front page lists the texts of all essential hazard/precautionary-phrases (H/P-phrases) or risk/safety-phrases (R/S-phrases) from all substances. The back page possesses a large section (4) for additional notes to make the user aware of particular rules regarding dangers, safety rules, behaviors in case of danger, and waste disposal. The final block (5) yields space for two signatures, usually one of the chemical user and one of the supervisor.

Still, one has to fill all these sections with content, which is the most laborious and most time consuming step. Theoretically, one can use any word processor for this task. Practically, one will miss handy features such as import and export functions for often used chemicals such as acetone, diethyl ether, sodium hydroxide, and others. Commercial software solutions [3] are available, but most of them are overloaded with features, use proprietary file formats, and are very expensive. On the other hand, there are some free online tools such as GisChem [4], but they require permanent online access, and do not allow to create local databases (or no databases at all), which makes managing data very difficult.

This situation prompted us to write a program for creating, managing and printing such group safety data sheets. It was released as a free tool and is described in this article. Initially, we used ‘Beryllium’ as a working title for this spare time project. However, the first version written in C++/WTL (Windows Template Library, see [5]) spread very fast and so the name remained. In 2012, a complete rewrite was done in C++/wxWidgets (see [6]) to provide a Linux version, too. It was called ‘Beryllium^10^’ because of the very long half-life of ^10^Be that makes it the second most stable isotope of this element [7].

## Implementation

### Design criteria

One has to accomplish the following tasks to create and apply a group safety data sheet: 

1. Create a list of required substances

2. Look for and download safety data for each substance

3. Compile, sort, and arrange data

4. Check and edit data if necessary

5. Save and print data

6. Brief oneself and/or others with the safety data

A list of substances is automatically achieved when planning an experiment or process with chemicals involved. The last point cannot be implemented by any program since the chemical user always bears the responsibility to comply with safety rules, which includes briefing oneself and others of potential risks and safety precautions. This is confirmed by signature on the GSDS. Except for these two steps Beryllium^10^ will support the user with the remaining tasks. The following paragraphs describe the implementation of these.

The development of Beryllium^10^ followed some specific design criteria. First, the program should require no installation beyond file copying. Secondly, the program should provide an easy-to-understand, minimal, and therefore clear user interface, which allows to change most of the content by just clicking on it. Thirdly, data should be stored in an human-readable format. Last, the program should both support the old regulation system (R/S phrases, orange hazard symbols) [8] as well as the new *Globally Harmonized System of Classification and Labeling of Chemicals* (GHS, H/P phrases, red/white hazard symbols) [1,9]. Also, the inability to change the layout was designed on purpose for providing a uniform experience when working with the program and GSDSs.

### Collecting data

**Figure 2:**
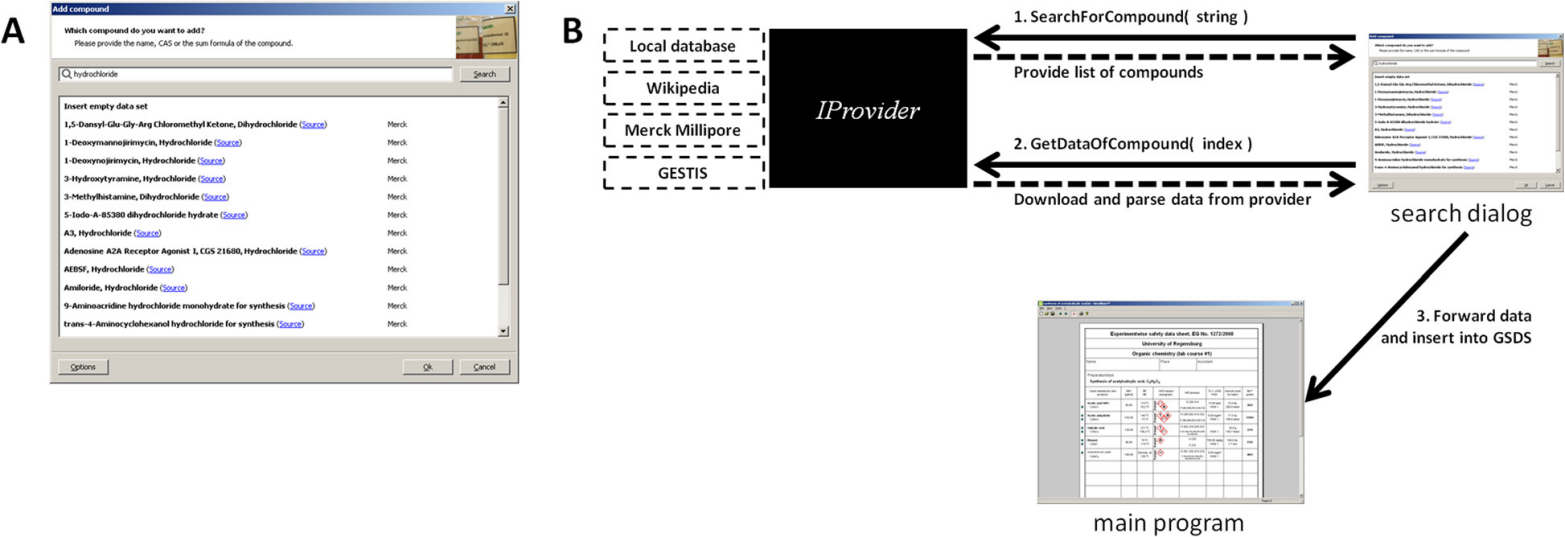
**Search dialog.A.** Screenshot of the search dialog showing results for ‘hydrochloride’. Each result provides at least the name of the compound, a link to the orginal source, and the provider name. Additional information depends on the provider itself. For instance, the local database reports the CAS registry number and the time of download as well. **B.** Communication of the search dialog with the provider interface. First, each provider (through the interface) is asked for a list of compounds for a given search string and the list is filled with the results. Secondly, when the user has chosen a compound and clicked ‘Ok’ on the dialog, the data set is downloaded from the corresponding provider and forwarded to the main program.

One of the core parts of Beryllium^10^ is the search dialog, which is opened when the user clicks on a blank line to add a new compound to the data sheet. This dialog is depicted in Figure 2A and provides two functions, viz. searching for a compound and subsequently downloading its safety data set. In the first step, the user enters either a (part of) name, CAS number, or sum formula of a compound to look for. The program then queries the available providers with the search string and fills the list with the responses. In a second step, the user selects one of the search results and the corresponding provider is requeried for the data of the compound selected. The data is inserted into the GSDS and, optionally, it can be saved to a local database chosen by the user for later reuse.

In this context, a provider is an abstract source of (safety) data. Internally, it is represented by the abstract interface class *IProvider*. This interface provides virtual functions for the communication with the search dialog and also implements helper functions used by all derivates. The specific providers are implemented in derivated classes. At the moment there are four providers available, viz. *CBerylliumProviderLocal*, *CBerylliumProviderWiki*, *CBerylliumProviderGESTIS*, and *CBerylliumProviderMerck*, which correspond to the local database, Wikipedia, GESTIS [10] and Merck Millipore [11], respectively. The search dialog just fills a vector with all available providers and queries them by the appropriate functions when needed. This design allows easy addition of new import sources to Beryllium^10^.

The actual implementation of the providers differ a lot in terms of complexity. *CBerylliumProviderLocal* only needs to load data from a structured XML file from the hard disk, whereas *CBerylliumProviderMerck* has to download and parse the complex and (partly) chaotic HTML output from the dynamic Merck Millipore webpage [11]. Although HTML is standardized [12], it is meant to be displayed in a browser. Browsers are very tolerant regarding incomplete, wrong, and missing tags, which are almost naturally generated by such a complex dynamic webpage. Therefore, these are not visible while surfing but can cause a lot of trouble when parsing. So, a lot of time is spent in this implementation not only for finding and extracting the wanted tables and data, but rather for ‘fixing’ the HTML output. Unfortunately, this also results in high effort in maintaining code and is the reason for rather infrequent updates on this part of Beryllium^10^.

In the end and regardless of the method of data acquiring, the provider generates a filled data object, which is just copied and forwarded to the main program and inserted into the GSDS. The diagram in Figure 2B compiles and explains the communication between the interface (and its derived classes), the search dialog, and the main program.

It should be noted that the median lethal doses (LD50) are not read in because of the following issue. Identifying a value and its unit is simple. However, there are innumerable ways how vendors are providing the type of exposure and the associated species. They are given with or without brackets, or put before or behind the value. Multiple LD50 values are sometimes grouped together in one line, sometimes separately in multiple lines, and sometimes in tables. In some cases they are even given in a different language from the rest of the MSDS. Even for the same vendor, formats are inconsistently applied. Due to this, parsing of LD50 values often results in a incomplete and inaccurate toxicological data set. This would force the user to manually check and compare the read in values with the original safety sheet, every time. So, it does not make any difference whether the toxicological data is downloaded automatically or transferred by hand. Consequently, the LD50 values have to be transferred by hand at the moment to avoid any problems. However, the user can easily open the original safety data sheet by clicking on the link provided on the ‘Source’ tab of the substance properties dialog to do this (see below).

## Result and discussion

### Managing data

Beryllium^10^ assists the chemical user in filling in and editing a GSDS. Some sections are fully or partially completed by the program itself. Naturally, other information such as the institute, name, or the task have to be manually entered by the user.

For instance, the ‘essential H/P phrases’ on the front page are automatically collected from all inserted substances and subsequently summarized. Of course, with more substances there are probably too many phrases to fit into the available space. Hence, the user can click on the block and decide, which phrases are more important (regarding the task) and should be shown, and which ones are less important and be hidden.

Similarly, the back page is partially filled in. First, the program inserts some standard phrases such as ‘*Wear suitable protective clothing, gloves and eye/face protection at all time*’. Secondly, substance-specific sentences are added. These are generated from both the ‘Hints’ page in the properties dialog of a substance and from phrase-dependent templates. For example, R17 would add ‘*Only work under inert gases.*’ to the ‘Protective measures and safety rules’-section. Templates can be modified by editing of ‘*templates_xx.xml*’ (xx is the language code) in the ‘config’-subdirectory. Substance-specific sentences are added with the preceded, bold substance name. Once again, the user can again click on one of the sections on the back page to edit or add general sentences.

**Figure 3:**
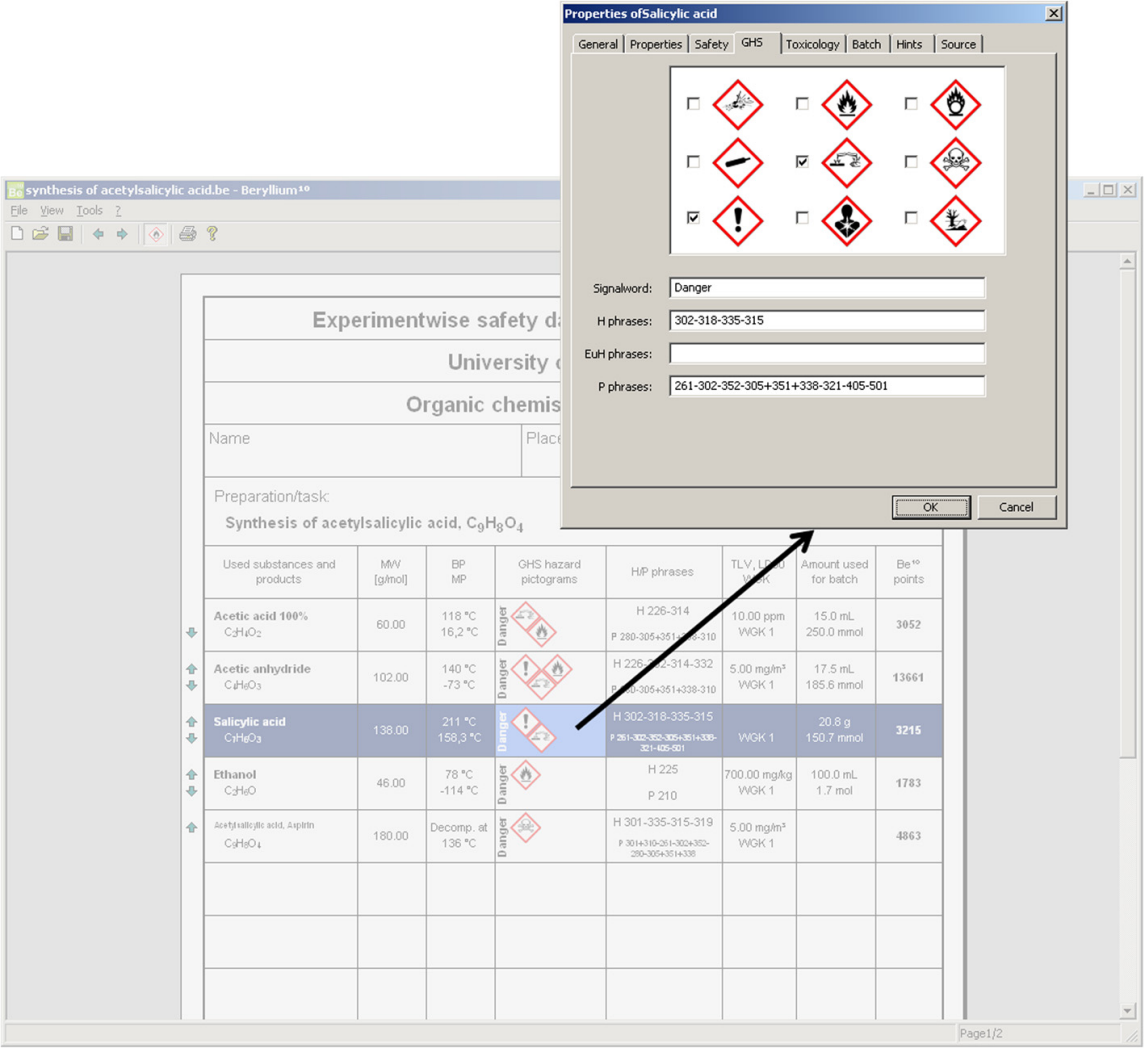
Screenshot of the main window and substance properties dialog.

The main part of a GSDS is the substance block on the front page. The user is able to add a new entry by clicking on a blank line in this block. This will open the search dialog, which is described in the previous section. Although, there is only space for nine substances on one front page, Beryllium^10^ will automatically add a new couple of pages (with the same head sections), if this limit is reached. Clicking on an entry opens the properties dialog, which allows to modify all available data about an entry. The tab shown, when the dialog is opened, depends on the column that was selected while clicking. Figure 3 shows the dialog and also the main window of Beryllium^10^. This dialog gives not only access to all visible data on the sheet but also to some meta data, such as the CAS number, the density, and the source (including link and time of download), and modification date of the entry. Although not visible, these properties are used for managing the data in the program. The CAS number is used for comparing when exporting an entry to a library, the density is needed for calculation of concentration and amount of substance, and the source data gives the user the opportunity to check and compare the imported data with the original.

**Figure 4:**
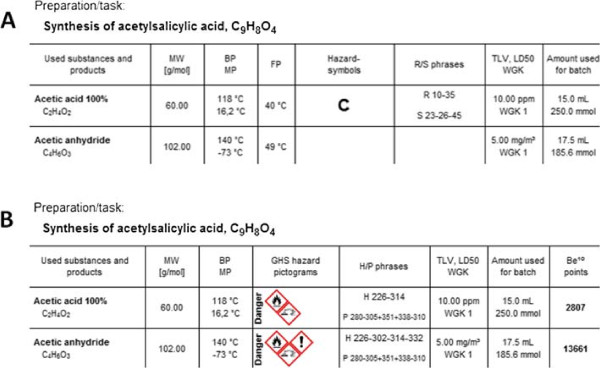
Two examples (A and B) for different sets of columns in the substance block.

The visible data is compiled and tabulated in columns within an entry line. Since not all information is always useful and needed for every task, the columns visible in the substance block can be customized by clicking on the head of the table. Figure 4 displays two examples, which together present all available columns. Most of the columns are self-explanatory. The first columns display the name, sum formula, molecular weight (MW), melting point (MP), boiling point (BP), and the flash point (FP).

Since Beryllium^10^ supports both the old and new hazard symbols and phrases, there are also columns available for both. The user can switch between them easily by clicking on a button on the toolbar of the main window or by selecting the respective columns. However, one should notice that the old system is no longer supported by all providers and therefore they may not provide any data for this anymore. In Figure 4A the data for acetic anhydride is missing because of this reason.

Toxicological data is presented in its own column with two lines. The first line is a limit concentration (c^limit^) and the second one shows the German water hazard class (WGK). There is a famous proverb, which should be considered in dealing with toxicological substances: ‘*Better be safe than sorry*’. Hence, Beryllium^10^ always selects the minimum concentration out of all available data of a substance for the first line. This can be the threshold limit value (TLV, or MAK in Germany) or one of the median lethal doses (LD50). These values are mostly given in units per volume or units per (body) mass depending on the most probable type of exposure to the substance. It is easy to compare concentrations of the same type but not of different ones. Therefore, reasonable reference systems are required. In the sense of the proverb mentioned above we decided to use a reference body mass of 50 kg (a small person) and a reference volume of 1.5 m^3^, which corresponds to the volume of a non-functional fume hood. The amount of substance can then be calculated using these reference systems and subsequently compared. The German water hazard class classifies substances into four categories from substances, which are not hazardous to water (class 0 or NWG) to substances, which are substantially hazardous to water (class 3). On the GSDS only the class number (1-3, or ‘-’ for class 0) is shown.

Finally, the last two columns show the entered batch and the so-called Be^10^-Points, respectively. The latter one are calculated by the program and explained in its own section (see below).

### Data format and printing

All data of a data sheet can be saved into one XML file [13]. This human readable format allows the import and export from and to other programs without any problems. Another plus is the expandability of this format, which allows to add other information (e.g. structural data). The overall structure of an Beryllium^10^-XML file is very straightforward and can be learned by just loading a file in a text editor.

The core element is the *substance*-element, which represents a complete entry from the substance block of a GSDS. The following shows an example, reduced *substance*-element for acetic anhydride:

A whole safety data sheet is mainly an array of these *substance*-elements plus some extra elements, which hold the texts for the head section, the texts for the back page, and the columns and phrases to show or hide. It is possible to export and import individual compound data to library files, which is just an array of *substance*-elements. If the library already contains an entry with the same CAS number, it will be replaced. A library file also can be used as a source (provider) for collecting data (see above).

Of course, the data sheet can be printed onto any printer (or in a PDF, if a PDF printer is available) that is supported by the operating system. Printing is required for signing the data sheet since digital signatures are not provided by Beryllium^10^ and are also not within the idea of a GSDS (i.e. audited safety information at hand).

### Be^10^-Points

Be^10^-points should give the chemical user a relative - NOT absolute - rating of the dangers of substances for the user. They are calculated on the basis of the available safety data of a compound. In general, these points are a sum of three terms, i.e. A, B, and C, which depend on the GHS pictograms and signal word, on the H- and P-statements, and on the batch, respectively.

For term A, a basic score was assigned to every GHS pictogram. This basic score may be modified by other physical or toxicological data. For instant, the pictogram 09 (‘Environmentally Damaging’) has a basic score of 100 and is multiplied by the water hazard class increased by one. Additionally, the signal word ‘Danger’ doubles the end score. All scores and the modifiers are listed in Table 1. Similarly, a score between 0 and 1000 was assigned to every H/P phrase. Term B just sums up these points. They can be viewed and modified by opening ‘*hazards_xx.xml*’ and ‘*precaution_xx.xml*’ (xx is the language code) in the ‘*config*’-subdirectory.

**Table 1:** Basic score and modifiers for calculation of term A

**No.**	**GHS pictogram (description)**	**Basic score**	**Modifier**
01	Explosive (exploding bomb)	300	-
02	Flammable (flame)	100	Doubled, if flash point is below 100 °C
03	Oxidizing (flame over circle)	200	Doubled, if flash point is below 100 °C
04	Compressed Gas (gas cylinder)	50	-
05	Corrosive (corrosion on hand and surface)	100	-
06	Toxic (skull and crossbones)	500	-
07	Irritant (exclamation mark)	50	-
08	Health hazard (health hazard)	100	-
09	Environmentally damaging (fish and tree)	100	Multiplied by (WGK + 1)
	Every pictogram		Score is doubled, if signal word is ‘Danger’

A basic score was assigned to each GHS pictogram. This score may be altered by the listed modifiers. See text in section ‘Be^10^-Points’ for more details.

The last term C checks the batch for exceeding the minimal limit concentration (c^limit^), which is the lowest LD50 or TLV available for this substance (see ‘Managing data’). It is calculated by the function in Equation 1.

(1) $$C=\frac{1000}{1+e^{-0.1\cdot (x_{1})}}\cdot \frac{10}{1+e^{-0.009\cdot (x_{2})}}$$

(2) $$x_{1}=100\cdot R-75$$

(3) $$x_{2}=100\cdot R-500$$

(4) $$R=\frac{{\text{c}}^{\text{batch}}}{{\text{c}}^{\text{limit}}}$$

**Figure 5:**
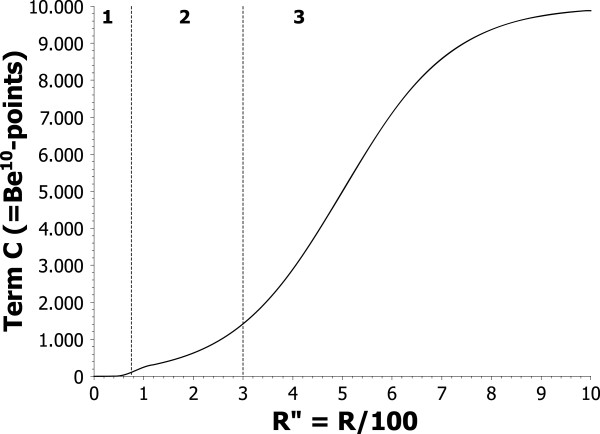
**Dependency of term C on R for calculating Be**^**10**^**-points.** The depicted plot shows the combination of two sigmoid functions to reflect the increasing danger with increasing ratio of batch concentration to the toxicological minimum limit concentration (e.g. TLV or LD50). In region 1 (i.e. working below the limit concentration) the plot starts very flat. The slope rises in region 2 (working at or a little over the limit concentration) and further rises strongly in region 3 (working far over the limit concentration) until it approaches a score of almost 10000 points at the 10-fold excess. See text in section ‘Be^10^-Points’ for more details.

From this equation it is clear that C is a combination of two sigmoid functions, which both ultimately depend on R. R is the ratio of the batch concentration and the minimal limit concentration (see Eq. 4). The construction of this particular function C followed specific criteria. First, the function should start very flat for R below 75%. Between 75% and 300% the function rises with a relative small slope to about 1500 points. After that the slope should strongly increase until the function reaches almost 10000 points for R equal 1000% (i.e. batch concentration is 10 times limit concentration). That behavior corresponds to the idea that working near and a little over the limit concentration is common for a professional chemist but great care must be taken if exceeding this limit too much. The function itself is plotted in Figure 5.

Obviously, the individual impact of each term on the total score differs and depend on the available data of a compound. For classified compounds with a small data set, i.e. one or two pictograms and only few (and general) H/P phrases, term A has a larger impact. This is reasonable since term A represents the GHS pictograms, which in turn represent very general warnings of dangers. On the other side, term B represents more accurate dangers and safety precautions. Therefore, it has the largest impact in most cases. Assuming a batch and corresponding toxicological data is provided, term C has usually a noticable impact on the total score, too. This is due to the fact that in many cases the batch will exceed at least one of the provided toxicological limit concentrations or doses (the ones for mice or rats) resulting in a high score for term C. Total scores for common chemicals and batches lie in between 1000 and 10000 points. However, term C dominates the total score and raises it over 10000 points if the batch exceeds the limit concentration too much.

It should be noted that this system makes no claims of being perfect and it may happen that dangers are over- or underestimated. It is a small approach to automatically estimate and compare dangers, and is provided as a support and sorting tool only. The calculation may change in future versions. The qualified chemical user always bears the responsibility!

### Single safety data sheet

Beryllium^10^ is also able to create single safety data sheets (SSDSs). In contrast to a GSDS, a SSDS possesses only the safety data for one compound on only one page. Nevertheless, these sheets are designed by the same basic principle, i.e. to have a clear overview of safety data at hand. SSDSs are useful in cases where one specific compound is continuously used. For instant, acetonitrile, methanol, and dichloromethane are common eluents in high–performance liquid chromatography (HPLC) [14]. SSDSs for these substances can be printed and put somewhere near or directly on the HPLC instrument for everyone to see. So, the safety information is always at hand.

## Conclusions

In this article we presented Beryllium^10^, a program for creating group safety data sheets. Group safety data sheets provide a short and clear overview of dangers and more for experiments and tasks, which are always at hand. Beryllium^10^ was developed to assist the user in the whole progress by providing a fast and clear user-interface, and well-conceived design for collecting and managing such safety data. It is already a popular tool at numerous universities in Germany, and we hope it becomes a useful tool at other institutes, universities and countries, too.

## Availability and requirements

Beryllium^10^ is publicly available software. It can be used, copied and distributed freely. A single zip-archive can be downloaded from the homepage, which contains both the Windows and the Linux version. The Windows version (beryllium10.exe) runs on Windows XP and 7, whereas the Linux version (uBeryllium10) runs on Ubuntu 12.04, Knoppix 7.2, and other Debian based distributions. They do not need any installation and, therefore, no administration rights to use. Since version 2.2 Beryllium^10^ is released under GNU GPL v3 and the source code is freely available on Sourceforge. This will especially allow other developers implementing new providers, and expand and adjust the functionality of the program. Also, it is possible to compile the program on other platforms than listed here. **Project name:** Beryllium^10^**Project homepage:**http://beryllium.keksecks.de**Sourceforge link:**http://sourceforge.net/projects/beryllium10/**Operating Systems:** Windows (XP, 7), Linux (Ubuntu, Knoppix and other Debian based distributions) **Programming language:** C++/wxWidgets **Other requirements:** No requirements **License:** GNU GPL v3 **Any restrictions to use by non-academics:** None

A current version of Berylium^10^ (Version 2.3, Build 1078) is available as ZIP–archive as Additional file 1. This archive contains both a compiled Windows and Linux binary, and all further files required to use the program together with some sample safety data sheets.

## Competing interests

The author declares that he has no competing interests.

## Supplementary Material

Additional file 1 **Berylium**^**10**^** Version 2.3, Build 1078.** This archive contains both the Windows and Linux binaries, and all further files required to use Beryllium^10^ together with some sample safety data sheets. Click here for file
