# Supplementary figures and images for: Beryllium10: a free and simple tool for creating and managing group safety data sheets
Source: J Cheminform. 2014 Mar 20;6:6. doi: 10.1186/1758-2946-6-6 (PMC3998190; doi:10.1186/1758-2946-6-6)

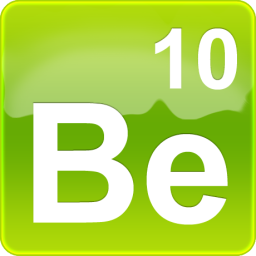

Supplement: Additional file 1 — Berylium10 Version 2.3, Build 1078. This archive contains both the Windows and Linux binaries, and all further files required to use Beryllium10 together with some sample safety data sheets. [file 1758-2946-6-6-S1.zip › config/beryllium10.png]
